# Perioperative Outcomes in Patients with and Without Chronic Preoperative Therapeutic Anticoagulation Undergoing Metabolic Surgery at an Academic Medical Center

**DOI:** 10.3390/jcm14020424

**Published:** 2025-01-10

**Authors:** Sami Fares, Juan S. Barajas-Gamboa, Kevin Zhan, Jerry T. Dang, Valentin Mocanu, Mélissa V. Wills, Gabriel Diaz Del Gobbo, Carlos Abril, Juan Pablo Pantoja, Alfredo Daniel Guerron, Javed Raza, Ricard Corcelles, John Rodriguez, Matthew Kroh

**Affiliations:** 1 Cleveland Clinic Lerner College of Medicine, Case Western Reserve University, Cleveland, OH 44106, USA; sxf306@case.edu (S.F.); dangj3@ccf.org (J.T.D.); abrilc@clevelandclinicabudhabi.ae (C.A.); corcelr@ccf.org (R.C.); rodrigj2@clevelandclinicabudhabi.ae (J.R.); 2 Department of General Surgery, Cleveland Clinic Abu Dhabi, Abu Dhabi 112412, United Arab Emirates; barajaj@clevelandclinicabudhabi.ae (J.S.B.-G.); gabodelgobbo@hotmail.com (G.D.D.G.); pantojj@clevelandclinicabudhabi.ae (J.P.P.); guerrod@clevelandclinicabudhabi.ae (A.D.G.); razaj@clevelandclinicabudhabi.ae (J.R.); 3 Cumming School of Medicine, University of Calgary, Calgary, AB T2N 1N4, Canada; kevin.zhan1@ucalgary.ca; 4 Department of General Surgery, Cleveland Clinic, Cleveland, OH 44195, USA; mocanuv@ccf.org (V.M.); willsm@ccf.org (M.V.W.)

**Keywords:** bariatric surgery, sleeve gastrectomy, Roux en-Y gastric bypass, therapeutic anticoagulation, complications

## Abstract

**Background/Objectives:** Patients on chronic anticoagulation undergoing metabolic surgery represent an increased risk of complications, including both bleeding and thrombotic events, such as deep vein thrombosis (DVT) and pulmonary embolism (PE). The optimal perioperative management of patients who are receiving chronic anticoagulation therapy (CAT) is complex. In the colorectal surgery literature, patients on CAT have a 10% rate of peri-procedural bleeding and a 3% rate of thromboembolism. The aim of this study was to evaluate and compare the safety and postoperative outcomes between patients with and without CAT undergoing Roux-en-Y gastric bypass (RYGB) or sleeve gastrectomy (SG) at a tertiary referral center in the United Arab Emirates (UAE). **Methods:** All patients who underwent primary bariatric surgery between September 2015 and July 2019 were retrospectively reviewed. The first group included patients with CAT, and the second group included patients without CAT. Demographics, perioperative outcomes, and postoperative results were examined. **Results:** Our study included 542 patients, 22 (4%) with CAT and 520 (96%) without CAT. Mean age was 46.3 ± 10.5 years in the CAT group and 36.0 ± 11.7 years in the non-CAT group (*p* < 0.001); median BMI was 41.8 (range 33.1–61.3) and 42.7 (range 30.1–78.4) kg/m^2^, respectively (*p* = 0.52). The CAT group had significantly higher rates of hypertension (77.2% vs. 32.5%, *p* < 0.001), obstructive sleep apnea (81.8% vs. 31.5%, *p* < 0.001), and coronary artery disease (31.8% vs. 2.8%, *p* < 0.001). In the CAT group, 8/22 (36.4%) patients underwent Roux-en-Y gastric bypass and 14/22 (63.6%) sleeve gastrectomy, compared to 228/520 (43.8%) and 292/520 (56.2%), respectively, in the non-CAT group (*p* = 0.51). There were no statistically significant differences in postoperative emergency department (ED) visits (18.1% vs. 24.2%, *p* = 0.51), early major complications (4.5% vs. 3.4%, *p* = 0.54), readmission rates within 30 days (4.5% vs. 3.6%, *p* = 0.56), or late complications (4.5% vs. 4.2%, *p* = 0.60). Mean length of stay was significantly longer in the CAT group (4.6 vs. 2.6 days, *p* < 0.001). The mean follow-up was 10 ± 7.3 months for the CAT cohort and 11 ± 9.7 months for the non-CAT cohort (*p* = 0.22). Weight loss outcomes at 12 months were comparable, with a percent total body weight loss (TBWL) of 27.0 ± 7.3% in the CAT group and 28.9 ± 8.3% in the non-CAT group (*p* = 0.29). There were no deaths in either group. **Conclusions:** In this series, at a tertiary referral center in the UAE, metabolic surgery is safe for CAT patients. Multidisciplinary preoperative preparation might be warranted to avert potential complications.

## 1. Introduction

Obesity has emerged as a global health crisis, with particularly alarming rates in the Middle East and North Africa (MENA) region. In the United Arab Emirates (UAE), the prevalence of obesity in adults reached 31.7% in 2016, significantly higher than the global average of 13% [1]. This epidemic has led to a surge in obesity-related comorbidities, including conditions requiring chronic anticoagulation therapy. As these comorbidities become more prevalent, the demand for effective treatment strategies, such as metabolic surgery, has grown accordingly.

Metabolic surgery, primarily Roux-en-Y gastric bypass (RYGB) and sleeve gastrectomy (SG) has become a cornerstone in the management of severe obesity [2]. However, the increasing prevalence of patients on chronic anticoagulation therapy (CAT) presents unique challenges in perioperative management. While patients on CAT are at risk of increased bleeding, bariatric surgery itself increases the risk of thrombotic postoperative complications, including deep vein thrombosis (DVT) and pulmonary embolism (PE) [3]. This dual risk makes the perioperative management of these patients particularly complex, requiring a delicate balance between preventing thrombosis and avoiding excessive bleeding [4].

In the MENA region, the use of oral anticoagulants has been rising steadily, with significant implications for bariatric surgery. Data from the Gulf Survey of Atrial Fibrillation Events (Gulf SAFE) registry indicate that 10.3% of patients with atrial fibrillation in the region are prescribed oral anticoagulants, while a UAE-specific study found that 8.7% of patients admitted to a large tertiary hospital were on chronic anticoagulation therapy [5,6]. This prevalence becomes particularly concerning in the context of bariatric surgery, where Western studies have demonstrated increased risks for anticoagulated patients. These risks include higher rates of postoperative complications (10.6% vs. 5.5% in non-anticoagulated patients), 30-day readmissions (9.2% vs. 5.0%), and perioperative bleeding complications (OR 2.81, 95% CI 1.80–4.38) [7,8].

However, comparable data for the MENA region, and specifically for the UAE, remain scarce. This paucity of evidence highlights a critical gap in our understanding of outcomes for anticoagulated patients undergoing bariatric surgery in the local context. The unique genetic, environmental, and lifestyle factors prevalent in this region may significantly influence both the risk profile and management strategies for these patients [9]. Furthermore, the distinct healthcare systems and clinical practices in the UAE may affect perioperative protocols and outcomes differently from those in Western populations.

This knowledge gap is particularly concerning given the rising rates of both obesity and conditions requiring chronic anticoagulation in the UAE [10]. As bariatric surgery continues to gain popularity as a treatment for severe obesity, understanding the specific risks and optimal management strategies for anticoagulated patients in this region becomes increasingly crucial. To address this urgent need for local, context-specific research, the objective of this study is to evaluate and compare the safety and postoperative outcomes between patients with and without CAT undergoing Roux-en-Y gastric bypass (RYGB) or sleeve gastrectomy (SG) at a tertiary referral center in the United Arab Emirates (UAE).

## 2. Methods

### 2.1. Study Design

A retrospective review of a prospectively maintained database was performed at an academic medical center in the Middle East from September 2015 to July 2019. This study was approved by our institution’s Research Ethics Committee under the internal number A-2017-029.

### 2.2. Population

**Figure 1:**
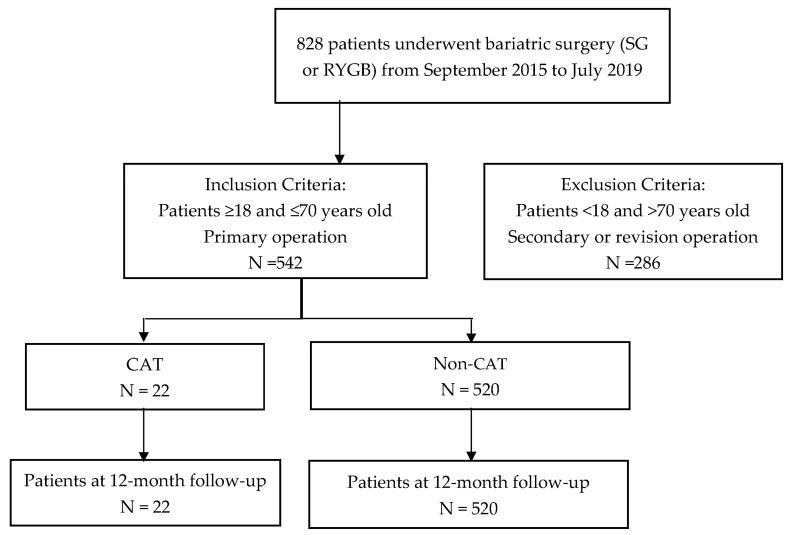
Study flow chart.

The patient population was comprised of two groups of patients that had undergone primary bariatric surgery, one subset with CAT and the other subset without. All patients were treated with either RYGB or SG (Figure 1).

### 2.3. Aims

The primary aim was to compare the perioperative and postoperative outcomes of patients with and without CAT undergoing primary bariatric surgery. The secondary aims were to compare the complication rates, conversion from laparoscopic to open surgery, and postoperative weight changes between the two groups.

### 2.4. Inclusion Criteria

All patients > 18 years of age undergoing primary bariatric surgery (RYGB or SG), with at least one year of follow-up data, with or without history of CAT, were included in this study.

### 2.5. Exclusion Criteria

All patients younger than 18 or greater than 70 years of age, patients undergoing a revisional or conversion procedure, patients without 1 year of follow-up data, procedures performed outside of the tertiary hospital previously mentioned, and patients undergoing endoscopic bariatric surgery procedures were not included in the study.

### 2.6. Preoperative Evaluation and Bridge Therapy

Preoperative workup included evaluation by our multidisciplinary team for patients undergoing primary bariatric surgery. Preoperative investigations comprised esophagogastroduodenoscopy (EGD), contrast-enhanced upper gastrointestinal series, and blood chemistry panels. Abdominal computerized tomography scans and/or abdominal ultrasounds were obtained at the discretion of the treating physicians.

For patients receiving CAT, bridge therapy was administered based on institutional protocol. For patients requiring bridge therapy, the following steps were taken prior to the procedure.

1. Stop warfarin therapy 5 days before surgery/procedure;
2. Begin low molecular weight heparin (LMWH) injections 24–48 h after stopping warfarin (start immediately if INR is sub-therapeutic or in patients with high thromboembolic risk);
3. Obtain INR on the day before or the day of the surgery/procedure;
4. If INR is > 1.7, consider the use of oral vitamin K;
5. Stop LMWH injections 12–24 h before surgery/procedure.

The preoperative bridging protocol was designed to balance the dual risks of perioperative bleeding and thrombosis. The 5-day warfarin cessation period was chosen to ensure adequate reduction in anticoagulation effects, as most patients achieve an INR <1.5 within 5 days of discontinuation. LMWH bridging was initiated 24–48 h after warfarin cessation to maintain adequate anticoagulation while minimizing the risk of drug interaction. The 12–24-h window for discontinuing LMWH before surgery was selected to allow sufficient clearance of anticoagulant effect while minimizing time without anticoagulation. For patients on direct oral anticoagulants (DOACs), the protocol was modified based on renal function and specific drug half-life. Vitamin K administration was reserved for cases with persistent elevation of INR to avoid over-reversal of anticoagulation. This protocol was developed in consultation with our hematology department and aligned with current international guidelines for perioperative anticoagulation management.

### 2.7. Intraoperative Management

#### Surgical Techniques

The choice between SG and RYGB was made based on patient-specific factors and surgeon discretion, with the final decision reached through shared decision-making.

SG: After standard patient identification and preoperative team briefing, the patient was positioned supine. Following aseptic preparation, a 5 mm optical trocar was inserted in the left upper quadrant to establish pneumoperitoneum. Bilateral transversus abdominis plane blocks were administered. Additional trocars were placed in a U-shaped configuration. A Nathanson liver retractor was positioned under direct visualization.

The procedure began with the dissection of the phrenoesophageal fat pad to expose the angle of His. The greater curvature was mobilized by dividing the gastrocolic ligament, stopping approximately 5 cm proximal to the pylorus. Short gastric vessels were divided to fully mobilize the fundus. With a 40 French Bougie in place, the sleeve was created using sequential firings of a linear stapler. Hemostasis was confirmed, and the resected stomach was removed. The trocar site was closed with a figure-of-eight #0 Vicryl suture. Endoscopy was performed to confirm sleeve patency and the absence of leaks. After removing the liver retractor and releasing the pneumoperitoneum, skin incisions were closed with 4–0 Monocryl.

RYGB: Following similar patient preparation and trocar placement as in SG, the procedure commenced with the identification of the left gastric pedicle. The descending branch was transected just distal to this point using a reinforced purple load stapler. A small gastric pouch was created using multiple firings of an endo-GIA stapler. The ligament of Treitz was identified, and the jejunum was transected 100 cm distally using a GIA stapler with a tan load. A 100 cm Roux limb was measured, and a side-to-side jejunojejunostomy was created using a 60 mm GIA stapler with a tan load. The enterotomy was closed with a running 2–0 Vicryl suture. The mesenteric defect was closed with 2–0 Ethibond, followed by omental division.

The gastrojejunostomy was constructed using a linear stapler, and the Pseudo-Petersen’s defect was closed with a running 2–0 Ethibond suture. All 12 mm trocar sites were closed using #0 Vicryl with the Carter-Thomason device. Endoscopy was performed to confirm anastomotic patency and the absence of bleeding or leakage. After removing the liver retractor and releasing the pneumoperitoneum, skin incisions were closed with 4–0 Monocryl.

The choice between SG and RYGB was based on multiple factors, including the presence of gastroesophageal reflux disease (GERD), severity of diabetes mellitus, BMI, and patient preference. RYGB was generally preferred for patients with severe GERD, poorly controlled diabetes, or BMI > 50 kg/m^2^. SG was favored for patients with higher bleeding risk, including those on anticoagulation, given its technically simpler nature and reduced anastomotic complications. The final decision was reached through shared decision-making between the surgeon and patient after discussing the risks and benefits of each procedure.

### 2.8. Postoperative Care and Follow Up

Postoperatively, patients were admitted to the surgical ward under a standardized recovery protocol. DVT prophylaxis included early ambulation, sequential compression devices (SCDs) worn continuously until discharge, and chemoprophylaxis with LMWH. The protocol also included multimodal narcotic-sparing analgesia and initiation of a clear liquid diet the day after surgery. All patients were encouraged to ambulate within 4 h after surgery, with subsequent ambulation every 2 h while awake during their hospital stay. After monitoring for perioperative complications, patients were discharged to home when tolerating adequate oral intake. Patients follow up with evaluation in our outpatient clinic with members of our multidisciplinary team at intervals of 1 week, 1 month, and then every 6 months postoperatively for 3 years. Following our institutional protocol, patients were required to have an EGD and a fluoroscopy 12 months after surgery or, if necessary, before.

CAT patients received enhanced postoperative monitoring due to their increased risk for both bleeding and thrombotic complications. Specific protocols included the following:

- Hemoglobin checks every 6 h for the first 24 h, then daily until discharge;
- Daily PT/INR monitoring during bridging therapy;
- Clinical assessment for bleeding signs (tachycardia, hypotension, drain output) every 4 h for the first 48 h;
- Mechanical thromboprophylaxis with sequential compression devices maintained continuously until full mobilization;
- Early mobilization protocol starting 4 h post-surgery with subsequent ambulation every 2 h while awake;
- Daily assessment by both surgery and hematology services to adjust anticoagulation;
- Clear thresholds for imaging studies (CT scan or upper endoscopy) based on predefined clinical parameters;
- Specific discharge criteria including stable hemoglobin, therapeutic INR (for warfarin patients), and adequate oral intake.

For patients receiving CAT, bridge therapy was administered based on institutional protocol. For patients requiring bridge therapy, the following steps were taken following the procedure:

1. Start LMWH therapy 12–24 h after the surgery/procedure unless there are bleeding complications;
2. Start warfarin the day of or the day after the surgery/procedure unless there are bleeding complications;
3. Discontinue LMWH therapy when INR is within the therapeutic range.

LMWH bridging was continued postoperatively until therapeutic oral anticoagulation was achieved. For patients on warfarin, the target INR was typically reached within 5–7 days post-discharge, with daily INR monitoring through our anticoagulation clinic.

### 2.9. Surgical Outcomes

A comprehensive set of primary and secondary outcomes was assessed to evaluate the safety and efficacy of bariatric surgery in patients with and without CAT. These included length of hospital stay, unplanned intensive care unit admissions, emergency department visits, hospital readmissions, and reoperations within 30 days and 1 year postoperatively. Early complications (within 30 days) and late complications (30 days to 1 year) were documented, including bleeding events, anastomotic leaks, wound infections, and venous thromboembolism. Weight loss outcomes were evaluated using the percentage of total body weight loss and change in body mass index at 12 months post-surgery. Mortality rates, both perioperative and within the 1-year follow-up period, were also recorded. These outcomes were systematically compared between the CAT and non-CAT groups to assess the impact of chronic anticoagulation on bariatric surgery outcomes.

Postoperative complications were graded according to the Clavien–Dindo classification system:

- Grade I: Any deviation from normal postoperative course without need for pharmacological treatment or surgical, endoscopic, and radiological interventions.
- Grade II: Requiring pharmacological treatment.
- Grade III: Requiring surgical, endoscopic, or radiological intervention:
  - Grade IIIa: Intervention not under general anesthesia;
  - Grade IIIb: Intervention under general anesthesia.
- Grade IV: Life-threatening complication requiring IC/ICU management:
  - Grade IVa: Single-organ dysfunction;
  - Grade IVb: Multi-organ dysfunction.
- Grade V: Death of a patient.

### 2.10. Data Collection and Statistical Analysis

Data were collected retrospectively from electronic medical records and maintained in an institutional registry. The collected data encompassed patient demographics such as age, gender, weight, BMI, and ASA classification, along with comprehensive comorbidity information, including hypertension, hyperlipidemia, GERD, obstructive sleep apnea, diabetes mellitus, chronic kidney disease, coronary artery disease, COPD, and smoking status. Surgical details were documented, including procedure type (RYGB vs. SG), operative time, estimated blood loss, conversion rates, and intraoperative complications. For patients on anticoagulation, we recorded medication type and indication for therapy. Outcome measures included length of stay, postoperative complications (both early and late), ED visits, readmissions, reoperations, and weight loss outcomes (%TBWL). Detailed descriptive statistics were calculated for all variables in the study.

The Shapiro–Wilk test was used to assess the normality of continuous variables. Based on Shapiro–Wilk testing, age, operative time, and %TBWL followed normal distributions and were, therefore, analyzed using parametric tests (independent *t*-tests) and reported as means ± standard deviations. BMI, length of stay, and estimated blood loss showed non-normal distributions and were analyzed using non-parametric tests (Mann–Whitney U) and reported as medians with ranges. For categorical variables such as gender, comorbidities, and complications, Chi-square tests were used, with Fisher’s exact test applied when expected cell counts were less than 5. For comparisons of baseline characteristics between groups, we calculated standardized mean differences (SMD) alongside *p*-values. SMD values of 0.2, 0.5, and 0.8 were considered to represent small, medium, and large effect sizes, respectively. To compare outcomes between the CAT and non-CAT groups, we employed both parametric and non-parametric methods as appropriate. A *p*-value < 0.05 was established as the threshold for statistical significance across all analyses. To account for multiple testing, a Bonferroni correction was applied where applicable. All statistical analyses were performed using R software (version 2.13, The R Foundation for Statistical Computing, Vienna, Austria).

### 2.11. Definitions

CAT: Defined as the use of oral anticoagulants for at least 1 month before surgery with a minimum course of 6 months of continuous use.

Bridge Therapy: Defined as the use of short-acting anticoagulants (most commonly heparin or LMWH) for a period of time during interruption of warfarin therapy when the INR is not within a therapeutic range.

Major complications: any complication that results in a prolonged hospital stay (>7 days), reoperation, or reintervention

Minor complications: any complication not considered major, such as transient nausea/vomiting, urinary tract infection, etc.

Percentage of total body weight loss (%TBWL): calculated as (preoperative weight − follow-up weight)/preoperative weight × 100.

## 3. Results

### 3.1. Demographic Characteristics

A total of 542 patients were included in the study, with 22 patients (4.1%) in the CAT cohort and 520 patients (95.9%) in the non-CAT cohort. The CAT cohort was significantly older on average, with a mean age of 46.3 ± 10.5 years, whereas the non-CAT cohort had a mean age of 36.0 ± 11.7 years (*p* < 0.001). The initial median BMI for the CAT cohort was 41.8 kg/m^2^ (range 33.1–61.3), while for the non-CAT cohort, it was 42.7 kg/m^2^ (range 30.1–78.4), showing no significant difference (*p* = 0.52). Gender distribution was similar, as the CAT cohort had 59.1% females, and the non-CAT cohort had 61.7% females (*p* = 0.80).

The CAT group had several common thrombotic and cardiovascular conditions that served as indications for anticoagulation therapy, including deep vein thrombosis (31.8%), atrial fibrillation (31.8%), and coronary artery disease (22.7%). Less frequent indications included peripheral vascular disease, Buerger disease, and cerebrovascular accidents, each accounting for 4.5% of cases. In terms of anticoagulation therapy, enoxaparin and rivaroxaban were the most frequently used, each in 27.3% of patients. Apixaban was prescribed in 18.2% of cases, while warfarin and dabigatran were used in 13.6% of patients each.

Importantly, the CAT cohort exhibited a higher prevalence of several comorbidities. Hypertension was significantly more common in the CAT group (77.2% vs. 32.5%, *p* < 0.00), as was obstructive sleep apnea (81.8% vs. 31.5%, *p* < 0.00), coronary artery disease (31.8% vs. 2.8%, *p* < 0.00), and chronic obstructive pulmonary disease (9.0% vs. 1.1%, *p* = 0.003). The CAT cohort also showed higher rates of hyperlipidemia (63.6% vs. 45.3%, *p* = 0.09), gastroesophageal reflux disease (36.3% vs. 24.8%, *p* = 0.22), type 2 diabetes mellitus (40.9% vs. 29.2%, *p* = 0.24), and chronic kidney disease (13.6% vs. 4.6%, *p* = 0.05), although these differences did not reach statistical significance. The rate of current smokers was similar between the two groups (13.6% CAT vs. 14.6% non-CAT, *p* = 0.90). Additional details of comorbidities are described in Table 1.

### 3.2. Intraoperative Details

No statistically significant differences were observed in intraoperative findings between the CAT and non-CAT cohorts. The distribution of surgical techniques was similar between the two groups (*p* = 0.51), with 36.4% of the CAT cohort and 43.8% of the non-CAT cohort undergoing Roux-en-Y gastric bypass (RYGB), while 63.6% of the CAT cohort and 56.2% of the non-CAT cohort underwent sleeve gastrectomy (SG). The average operative time was comparable between the two groups, with a mean procedure length of 126 ± 57.9 min for the CAT cohort and 122 ± 48.4 min for the non-CAT cohort (*p* = 0.68). All cases in both cohorts were initiated laparoscopically, with only one conversion to open approach in the non-CAT group (0.2%), while no conversions were required in the CAT group.

Intraoperative complications (IOCs) were rare, occurring in 0% of cases in the CAT cohort and 0.7% of cases in the non-CAT cohort. Notably, there were no intra-operative blood transfusions required in either group. Estimated blood loss was minimal (< 25 mL) in all cases for both cohorts (Table 2).

### 3.3. Perioperative Outcomes, Early and Late Complications

The median length of stay was significantly longer in the CAT cohort compared to the non-CAT cohort (4.6 days vs. 2.6 days, *p* < 0.00). However, there were no statistically significant differences in complication rates between the two cohorts. Early minor complications occurred in 1 (4.5%) patient in the CAT cohort and 12 (2.3%) patients in the non-CAT cohort (*p* = 0.41). The most common minor complication was nausea and vomiting, occurring in 1 (4.5%) patient in the CAT cohort and 3 (0.6%) patients in the non-CAT cohort (*p* = 0.12).

Early major complications were observed in 1 (4.5%) patient in the CAT cohort and 18 (3.4%) patients in the non-CAT cohort (*p* = 0.54). Notably, there were no cases of venous thromboembolism (VTE) or gastrointestinal (GI) hemorrhage in the CAT cohort, while the non-CAT cohort experienced 1 (0.2%) case of VTE and 10 (1.9%) cases of GI hemorrhage. The rates of anastomotic leaks were extremely low, with no cases in the CAT cohort and 2 (0.35%) cases in the non-CAT cohort. One case of cerebrovascular accident (CVA) occurred in each cohort, representing 4.5% of the CAT group and 0.2% of the non-CAT group (*p* = 0.06).

Late complications were similar between the two groups, occurring in 1 (4.5%) patient in the CAT cohort and 22 (4.2%) patients in the non-CAT cohort (*p* = 0.60). The most common late complication in the non-CAT group was anastomosis stricture (1.8%), while the CAT group experienced one case (4.5%) classified as “other” (*p* = 0.18). Detailed information describing perioperative complications is included in Table 3.

### 3.4. Follow-Up Outcomes

The mean follow-up was 10 ± 7.3 months for the CAT group and 11 ± 9.7 months for the non-CAT group (*p* = 0.22). Analysis of postoperative outcomes revealed no statistically significant differences between the CAT and non-CAT cohorts (Table 4). Emergency department (ED) visits were slightly less frequent in the CAT group (18.1%) compared to the non-CAT group (24.2%), but this difference was not statistically significant (*p* = 0.51). Readmission rates within 30 days of surgery were similar, with 4.5% for patients in the CAT cohort and 3.6% for patients in the non-CAT cohort (*p* = 0.56). There were no readmissions after 30 days in the CAT group, while the non-CAT group had a 2.8% readmission rate beyond 30 days postoperatively.

Weight loss outcomes at 12 months postoperatively were comparable between the two groups. The mean BMI at 12 months was nearly identical, with 31.8 ± 7.1 kg/m^2^ in the CAT cohort and 31.7 ± 6.9 kg/m^2^ in the non-CAT cohort (*p* = 0.94). The percentage of TBWL at 12 months was slightly lower in the CAT group (27.0 ± 7.3%) compared to the non-CAT group (28.9 ± 8.3%), but this difference was not statistically significant (*p* = 0.29). Notably, there were no mortalities in either cohort throughout the study period (Table 4).

## 4. Discussion

Our study demonstrates that metabolic surgery can be performed safely in patients on CAT, with outcomes comparable to those not on anticoagulation. Despite the CAT group having a significantly higher prevalence of comorbidities, including hypertension, obstructive sleep apnea, and coronary artery disease, we observed no significant differences in major postoperative complications, readmission rates within 30 days, or weight loss outcomes at 12 months (%TBWL). The only significant difference was a longer length of stay in the CAT group, likely attributable to the need for careful anticoagulation management in the immediate postoperative period.

Different previous studies with varying cohort sizes and patient characteristics have reported on the safety of bariatric surgery in anticoagulated patients, with which our findings are aligned. Gerin et al. [11] conducted a case-matched study of sleeve gastrectomy that included 15 CAT patients and 30 matched controls. Their CAT cohort had similar characteristics to ours in terms of age (48.2 years) and BMI (44.2 kg/m^2^), though their sample size was smaller. They found no statistically significant differences in postoperative complications among anticoagulated patients. The major complication rates were 13.3% in the SG-CAT group and 3.3% in the SG-control group (*p* = 0.21), with one case of postoperative bleeding reported in each group (6.7% and 3.3%, *p* = 0.62). The incidence of revisional surgery was 13.3% in the SG-CAT group compared to 3.3% in the SG-control group (*p* = 0.21). Similarly, a study by Abi Mosleh et al. [12] analyzed a larger cohort of 84 CAT patients (mean age 52.3 years, mean BMI 45.6 kg/m^2^) undergoing bariatric surgery, providing a more robust sample size than our study. They reported comparable rates of acute complications in CAT patients undergoing bariatric surgery, including gastrointestinal bleeding, which occurred in 3.6% of patients in the RYGB group compared to 4% in the SG group. Non-gastrointestinal bleeding complications were observed in 1.2% of patients in the RYGB group, while no cases were reported in the SG group.

Our findings of comparable weight loss outcomes between CAT and non-CAT groups (%TBWL of 27.0 ± 7.3% vs. 28.9 ± 8.3%, *p* = 0.29) align with the limited available literature on this topic. Gerin et al. [11] similarly found no significant differences in weight loss outcomes between anticoagulated and non-anticoagulated patients following sleeve gastrectomy, reporting comparable percentage weight loss (31% vs. 26.6%, *p* = 0.20) and excess weight loss (64% vs. 52%, *p* = 0.12) at 12 months. The recently published series by Abi Mosleh et al. [12] further supports these findings, demonstrating that CAT patients can achieve satisfactory weight loss despite their higher-risk profile. This consistent achievement of weight loss goals across studies is particularly noteworthy given that CAT patients often have multiple comorbidities that might theoretically impact their ability to engage in physical activity or comply with dietary recommendations. Our results suggest that the presence of chronic anticoagulation should not be considered a barrier to achieving satisfactory weight loss outcomes after bariatric surgery.

These results highlight the importance of individualized risk assessment and multidisciplinary care in the successful management of anticoagulated patients undergoing bariatric surgery. However, our results contrast with those of some earlier studies. For instance, Howell et al. [13] reported a higher incidence of postoperative complications in bariatric surgery patients on CAT (10.7%). Similar findings were reported by Modasi et al. [14], who observed significantly higher 30-day complication rates in the CAT group compared to the non-CAT group (8.7% vs. 3.4%, *p* < 0.00) and increased mortality rates in the CAT group compared to the non-CAT group (0.55% vs. 0.08%, *p* < 0.00). Our study did not observe this increased risk, possibly due to our stringent perioperative anticoagulation management protocol. This discrepancy might be explained by differences in patient populations and perioperative management strategies.

The longer hospital stay observed in our CAT group (4.6 vs. 2.6 days, *p* < 0.00) is consistent with findings from Altieri et al. [8], who reported extended hospitalizations for anticoagulated patients undergoing bariatric procedures (ratio 1.2, 95% CI 1.199–1.241, *p* < 0.00). This increased length of stay is likely due to the need for careful monitoring and adjustment of anticoagulation therapy in the immediate postoperative period. Regarding weight loss outcomes, our findings of comparable %TBWL at 12 months between the CAT and non-CAT groups (27.0% vs. 28.9%, *p* = 0.29) are consistent with those reported by Gerin et al. [11], who also found no significant difference in weight loss between anticoagulated and non-anticoagulated patients following bariatric surgery, including percentage weight loss (31% vs. 26.6%, *p* = 0.20) and excess weight loss (64% vs. 52%, *p* = 0.12) at 12 months, respectively.

Our study also contributes to the growing body of evidence supporting the safety of bariatric surgery in high-risk populations. Notably, Pané et al. [15] demonstrated the safety and efficacy of bariatric surgery in patients with end-stage renal disease, another high-risk group. Similarly, Barajas-Gamboa et al. [16] reported favorable outcomes in elderly patients undergoing bariatric surgery, further reinforcing the notion that, with proper patient selection and perioperative management, bariatric surgery can be safely extended to various high-risk groups.

Our analysis suggests that strategies to optimize the length of stay in CAT patients undergoing metabolic surgery warrant further investigation [17,18]. The prolonged hospital stays observed in our cohort were primarily associated with patients on warfarin therapy who required careful bridging and INR monitoring. Modern approaches to reduce length of stay could include early hematology consultation for pre- and postoperative bridging management, with scheduled 1–2-week follow-up to ensure optimal drug levels after anticoagulation restart. Transitioning suitable patients from warfarin to modern DOACs could potentially reduce hospital stays, as these agents generally require less complex perioperative management. Historical practices often involved keeping patients hospitalized until therapeutic warfarin levels were achieved during LMWH bridging. Contemporary protocols now facilitate earlier discharge with outpatient LMWH bridging and daily INR monitoring until stable therapeutic levels are reached, coordinated through a multidisciplinary approach [19,20]. Implementation of such protocols, along with preoperative hematology consultation and standardized post-discharge monitoring pathways, could help optimize resource utilization while maintaining safety in this high-risk population.

Our study also contributes to the growing body of evidence supporting the safety of bariatric surgery in high-risk populations. Notably, Pané et al. [15] demonstrated the safety and efficacy of bariatric surgery in patients with end-stage renal disease, another high-risk group. Similarly, Barajas-Gamboa et al. [16] reported favorable outcomes in elderly patients undergoing bariatric surgery, further reinforcing the notion that, with proper patient selection and perioperative management, bariatric surgery can be safely extended to various high-risk groups. The increasing use of DOACs in surgical settings has also shown promising results. Recent evidence suggests that DOACs may offer advantages over traditional anticoagulation in terms of predictable pharmacokinetics and reduced monitoring requirements [21]. For instance, Kröll et al. [20] demonstrated the efficacy and safety of rivaroxaban for postoperative thromboprophylaxis in bariatric patients, while Leong et al. [19] provided comprehensive evidence supporting DOAC use after bariatric surgery. These findings, combined with our experience using DOACs in 59.1% of our CAT cohort (rivaroxaban 27.3%, apixaban 18.2%, dabigatran 13.6%), suggest that modern anticoagulation strategies may further improve the safety profile of bariatric surgery in high-risk patients.

Our study has several limitations. First, we did not control for differences in comorbidities between groups through propensity score matching or multivariate regression analysis, which could have helped account for the significantly higher prevalence of cardiovascular and respiratory comorbidities in the CAT group. Most notably, the CAT group was significantly older (mean age 46.3 vs. 36.0 years, *p* < 0.001), which may confound several of our findings. The higher prevalence of comorbidities such as hypertension (77.2% vs. 32.5%, *p* < 0.001), obstructive sleep apnea (81.8% vs. 31.5%, *p* < 0.001), and coronary artery disease (31.8% vs. 2.8%, *p* < 0.001), as well as the increased length of stay in the CAT group, could be partially attributed to this age difference rather than anticoagulation status alone. While our results showed comparable safety outcomes between groups despite these baseline differences, a matched analysis would have provided more robust evidence of equivalence. Second, there was a considerable sampling size imbalance between the CAT (n = 22) and non-CAT (n = 520) groups, which may affect the statistical power of our comparisons. This is particularly relevant for the analysis of complications, where the small size of the CAT group means that even a single event (or lack thereof) can dramatically affect percentages and potentially mask true differences between groups. For example, in our data, we observed no cases of VTE, GI hemorrhage, or anastomotic leaks in the CAT group, while these complications did occur in the non-CAT group at low rates (0.2%, 1.9%, and 0.35%, respectively). Conversely, the near-significant difference in CVA occurrence (4.5% vs. 0.2%, *p* = 0.06) based on a single event in each group illustrates how unbalanced group sizes can affect statistical comparisons of rare events. Third, our follow-up period was limited to 12 months, and longer-term outcomes remain to be evaluated. Fourth, while some might question the applicability of data from 2015 given the evolution of anticoagulation therapy, it is important to note that our study included patients on DOACs such as rivaroxaban (27.3%), apixaban (18.2%), and dabigatran (13.6%), making our findings relevant to current practice. Despite these limitations, our study has notable strengths. It represents one of the few studies of bariatric surgery outcomes in anticoagulated patients in the MENA region, addressing a significant gap in the literature. Moreover, our comprehensive analysis of perioperative outcomes provides valuable insights into the safety of bariatric procedures in this high-risk population within the context of the UAE healthcare system.

The multidisciplinary team approach employed in our center, involving close collaboration between bariatric surgeons, dietitians, anesthesiologists, hematologists, internal medicine and intensive care specialists, likely contributed to the favorable outcomes observed in our CAT patients. This approach is particularly relevant in the UAE and broader MENA region, where the prevalence of obesity and related comorbidities is high [22], and the need for safe and effective bariatric surgery in high-risk populations is growing. Future research should focus on longer-term follow-up and larger, prospective studies to confirm these findings. Additionally, investigating the optimal perioperative anticoagulation management strategies specific to bariatric surgery patients in the MENA region could further improve outcomes [23]. Studies exploring the cost-effectiveness of bariatric surgery in high-risk populations within the UAE healthcare system would also be valuable, especially considering the significant economic burden of obesity in the region [24].

## 5. Conclusions

In conclusion, our study demonstrates that metabolic surgery can be performed safely in patients on CAT in the UAE, with complication rates and weight loss outcomes comparable to non-anticoagulated patients. While these patients may require longer hospital stays, the benefits of bariatric surgery can be extended to this high-risk population with appropriate perioperative management.

## Figures and Tables

**Table 1:** Baseline characteristics and demographics.

Variables	CAT (N = 22)	Non-CAT (N = 520)	*p*-Values	Effect Size (SMD)
Age (mean +/− SD)	46.3 +/− 10.5	36 +/− 11.7	<0.00	0.92
Female (N, %)	13 (59.1)	321 (61.7)	0.80	0.05
Body weight (Kg, mean +/− SD)	119.7 ± 24.9	117.8 ± 23.8	0.72	0.15
BMI (median, kg/m^2^)	41.8 (33.1–61.3)	42.7 (30.1–78.4)	0.52	
ASA physical status classification ^‡^, median (min-max)	3 (2–3)	3 (1–4)	0.14	0.32
*Comorbidities (N, %)*				
Hypertension	17 (77.2)	169 (32.5)	<0.00	0.85
Hyperlipidemia	14 (63.6)	236 (45.3)	0.09	0.37
GERD	8 (36.3)	129 (24.8)	0.22	0.25
Obstructive sleep apnea	18 (81.8)	164 (31.5)	<0.00	0.89
T2DM	9 (40.9)	152 (29.2)	0.24	0.24
Chronic kidney disease	3 (13.6)	24 (4.6)	0.05	0.45
Coronary artery disease	7 (31.8)	15 (2.8)	<0.00	0.81
End-stage renal disease	0 (0.0)	4 (0.7)	0.68	0.12
COPD	2 (9.0)	6 (1.1)	0.00	0.48
Current smoker	3 (13.6)	76 (14.6)	0.90	0.03

Abbreviations: CAT, chronic anticoagulation therapy; BMI, body mass index; SD, standard deviation; ASA, American Society of Anesthesiologists; GERD, gastroesophageal reflux disease; T2DM, type 2 diabetes mellitus; COPD, chronic obstructive pulmonary disease. ^‡^ ASA physical status classification: 1 = healthy patient, 2 = mild systemic disease, 3 = severe systemic disease, 4 = severe systemic disease with constant threat to life.

**Table 2:** Intraoperative details.

Variables	CAT (N = 22)	Non-CAT (N = 520)	*p*-Values
*Surgical Technique (N, %)*			0.51
RYGB	8 (36.4)	228 (43.8)	
Sleeve Gastrectomy	14 (63.6)	292 (56.2)	
*Findings (N, %)*			
Conversions to open approach	0 (0)	1 (0.2)	-
Estimated blood loss < 25 mL	22 (100)	520 (100)	-
IOCs	0 (0)	4 (0.7)	-
Intra-operative blood Transfusion	0 (0)	0 (0)	-
Est. procedure length (min) (mean +/− SD)	126 +/− 57.9	122 +/− 48.4	0.68

Abbreviations: CAT, chronic anticoagulation therapy; SD, standard deviation; RYGB, Roux-En-Y Gastric Bypass; IOC, intra-operative complications.

**Table 3:** Perioperative outcomes, early and late complications *.

Variables	CAT (N = 22)	Non-CAT (N = 520)	*p*-Values
Length of stay (median, days)	4.6 (1–39)	2.6 (1–17)	<0.00
*Early minor complications (N, %)*	1 (4.5)	12 (2.3)	0.41
Nausea and vomiting (Grade I)	1 (4.5)	3 (0.6)	0.12
UTI (Grade II)	0 (0)	2 (0.3)	-
Other (Grade II–I)	0 (0)	3 (0.6)	-
*Early major complications (N, %)*	1 (4.5)	18 (3.4)	0.54
VTE (Grade II)	0 (0)	1 (0.2)	-
Anastomosis leakage (Grade IIIb)	0 (0)	2 (0.35)	-
GI Hemorrhage (Grade II–IIIa)	0 (0)	10 (1.9)	-
SBO (Grade IIIb)	0 (0)	2 (0.35)	-
Anastomosis stricture (Grade IIIa)	0 (0)	1 (0.2)	-
Surgical site infection (Grade II)	0 (0)	4 (0.8)	-
Sepsis (Grade IVa)	0 (0)	1 (0.2)	-
CVA (Grade IVa)	1 (4.5)	1 (0.2)	0.06
*Late complications (N, %)*	1 (4.5) -	22 (4.2)	0.60
Chronic nausea and vomiting (Grade I)	0 (0)	6 (1.2)	-
Internal hernia (Grade IIIb)	0 (0)	2 (0.35)	-
Anastomosis stricture (Grade IIIa)	0 (0)	9 (1.75)	-
Vitamin or mineral deficiency (Grade II)	0 (0)	1 (0.2)	-
Other (Grade I–II)	1 (4.5)	4 (0.7)	0.18

Abbreviations: UTI, urinary tract infection; VTE, venous thromboembolism; SBO, small bowel obstruction; CVA, cerebrovascular accident. * Complications are graded according to the Clavien–Dindo classification system: Grade I: Deviation from normal course without need for interventions; Grade II: Requiring pharmacological treatment; Grade IIIa: Requiring intervention not under general anesthesia; Grade IIIb: Requiring intervention under general anesthesia; Grade IVa: Life-threatening complication with single organ dysfunction; Grade IVb: Life-threatening complication with multi-organ dysfunction; Grade V: Death.

**Table 4:** Follow-up outcomes.

Variables	CAT (N = 22)	Non-CAT (N = 520)	*p*-Values
Follow-up months (mean +/− SD)	10 +/− 7.3	11 +/− 9.7	0.22
ED visits	4 (18.1)	126 (24.2)	0.51
Readmission < 30 days	1 (4.5)	19 (3.6)	0.56
Readmission > 30 days	0 (0)	15 (2.8)	-
BMI at 12 months (mean +/− SD)	31.8 +/− 7.1	31.7 +/− 6.9	0.94
%TBWL at 12 months (mean +/− SD)	27.0 +/− 7.3	28.9 +/− 8.3	0.29
Death	0 (0%)	0 (0)	-

Abbreviations: CAT, chronic anticoagulation therapy; ED, emergency department; BMI, body mass index; SD, standard deviation; %TBWL, percentage total body weight loss.

## Data Availability

The data that support the findings of this study are available from the corresponding author upon reasonable request.
